# Central Effects of 3-Iodothyronamine Reveal a Novel Role for Mitochondrial Monoamine Oxidases

**DOI:** 10.3389/fendo.2018.00290

**Published:** 2018-06-06

**Authors:** Annunziatina Laurino, Elisa Landucci, Laura Raimondi

**Affiliations:** ^1^Section of Pharmacology, Department of Neurology, Psychology, Drug Sciences and Child Health, University of Florence, Florence, Italy; ^2^Section of Clinical Pharmacology and Oncology, Department of Health Sciences, University of Florence, Florence, Italy

**Keywords:** 3-iodothyronamine, 3-iodothyroacetic acid, histamine, trace amine-associated receptors, monoamine oxidases

## Abstract

3-Iodothyronamine (T1AM) is the last iodinated thyronamine generated from thyroid hormone alternative metabolism found circulating in rodents and in humans. So far, the physiopathological meaning of T1AM tissue levels is unknown. Much is instead known on T1AM pharmacological effects in rodents. Such evidence indicates that T1AM acutely modifies, with high potency and effectiveness, rodents’ metabolism and behavior, often showing inverted U-shaped dose–response curves. Although several possible targets for T1AM were identified, the mechanism underlying T1AM behavioral effects remains still elusive. T1AM pharmacokinetic features clearly indicate the central nervous system is not a preferential site for T1AM distribution but it is a site where T1AM levels are critically regulated, as it occurs for neuromodulators or neurotransmitters. We here summarize and discuss evidence supporting the hypothesis that central effects of T1AM derive from activation of intracellular and possibly extracellular pathways. In this respect, consisting evidence indicates the intracellular pathway is mediated by the product of T1AM phase-I non-microsomal oxidation, the 3-iodothryoacetic acid, while other data indicate a role for the trace amine-associated receptor, isoform 1, as membrane target of T1AM (extracellular pathway). Overall, these evidence might sustain the non-linear dose–effect curves typically observed when increasing T1AM doses are administered and reveal an interesting and yet unexplored link between thyroid, monoamine oxidases activity and histamine.

## Background

3-Iodothyronamine (T1AM) is the last iodinated thyronamine produced by thyroid hormone (TH) metabolism and supposed to mediate TH non-genomic effects. From its identification in rodent tissues (1), T1AM was included within the family of the trace amines (TAs) and identified as an endogenous ligand for trace amine-associated receptors (TAARs), a family of constitutively active G protein-coupled receptors with species and tissue-specific expression cross-talking with the dopaminergic and the serotoninergic transmission (2, 3). This latter feature raised the interest around TAAR agonists, and then on T1AM, as potential treatments for controlling craving, pain, and neuropsychiatric illnesses. Consistently, the determination of endogenous T1AM brain levels or T1AM pharmacological administration would play diagnostic or therapeutic roles, respectively, in clinical manifestations of diseases involving dopamine release. However, after more than 10 years from T1AM discovery, whether central effects described following pharmacological administration of T1AM can be ascribed to TAAR activation remains an open issue, and we have no indications of T1AM levels in psychiatric or in drug-abusing patients. An important contribution to this uncertainness derives from the lack of pharmacological antagonists of TAARs with favorable pharmacokinetic features for *in vivo* administration. Instead, based on the current knowledge, T1AM presents a puzzling pharmacological profile suggesting a role as a cell messenger, behaving as a hormone and/or a neuromodulator, a function likely mediated also by 3-iodothryoacetic acid (TA1), the product of T1AM non-microsomal phase-I metabolism. T1AM may also interact with high-affinity targets including G protein-coupled receptors as TAAR1, or ion channels (4–6). We here summarize and discuss evidence indicating that behavioral effects of T1AM deserve to receive the attention of the investigators since, in the central nervous system, the pharmacokinetic features of this amine include a novel role for mitochondrial monoamine oxidases (MAO).

## T1AM Pharmacokinetic

### Few Notes on Tissue Distribution

Consisting evidence indicates that the tandem mass spectrometry represents the most reliable method to determine T1AM tissue levels, whereas some difficulties remain for measuring T1AM plasma levels (7) because T1AM administered to rodents circulated almost completely bound reversibly and with high affinity to Apo-B100, the main protein fraction of LDL and VLDL lipoproteins (8). This evidence indicated that T1AM has a specific plasma protein carrier, a condition usually reserved to hormones and vitamins and controlling amine pharmacokinetic.

**Figure 1:**
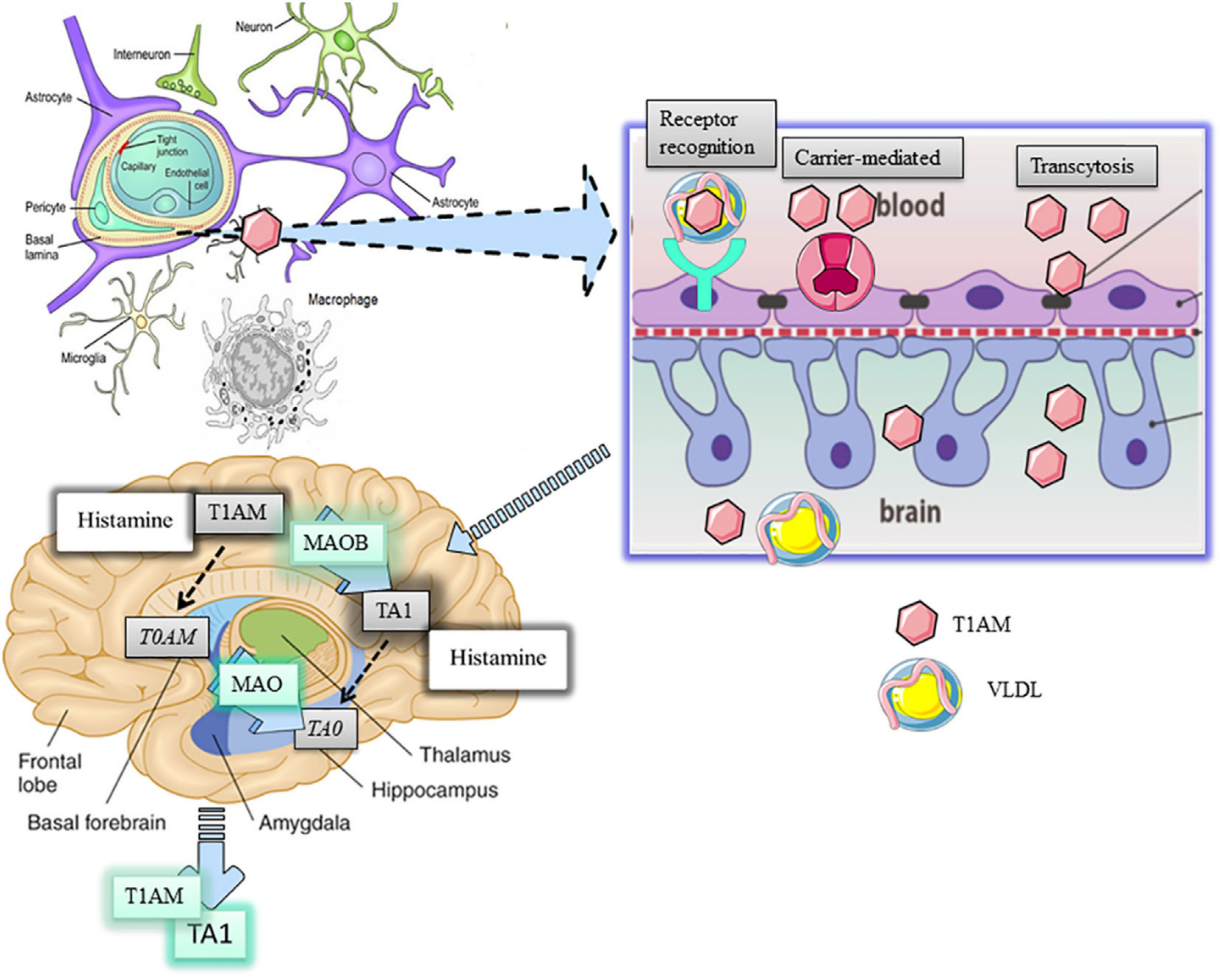
Distribution and metabolism of 3-iodothyronamine (T1AM) in the brain. T1AM is physiologically present in the brain of mice and its levels increse after pharmaoclogical administrtaion thus indicating T1AM can pass the blood brain barrier (BBB). The mechanism of T1AM passage through the BBB remains elusive yet. Three possible hypotheses are presented, including endocytosis of lipoprotein receptor, carrier-mediated transport, or transcytosis of free T1AM. One inside the brain, T1AM is manly metabolized to 3-iodothyroacetic acid (TA1) by the activity of mitochondrial monoamine oxidases (MAO).

LDL and VLDL are physiologically implicated in regulating the homeostasis of triglycerides and cholesterol, including their intracellular transport, by activating respective receptors (VLD-R and LDL-R), which are expressed almost ubiquitously but concentrated in the liver. Apo-B100 is the determinant for the recognition of lipoprotein receptors, their endocytosis, and recycling from plasma membrane. According to Roy et al. (8), the interaction of VLDL or LDL with their respective hepatic receptors represents a highly effective mechanism for the intracellular transport of T1AM. Once inside cells, T1AM, as well as cholesterol and triglycerides, may activate their own intracellular targets. In particular, since triglycerides may activate PPAR alpha while oxysterols the liver X receptor, genomic effects of T1AM may also represent epiphenomena derived from the activation of these targets (9, 10). Chiellini et al. (11) produced indications about T1AM distribution in mouse tissues. Following i.p. administration of [125I]-T1AM, the radioactivity recovered in tissues confirmed the liver and the gallbladder were preferential sites of T1AM distribution, reinforcing the link between T1AM, lipoproteins, and their receptor signaling, while the adipose tissue and skeletal muscle were described as sites where T1AM accumulated. Likewise, a small percentage of the radioactivity administered was recovered in the brain where such percentage could be even overestimated due to the accumulation of radioactive metabolites of T1AM. If we consider that circulating T1AM is almost completely bound to ApoB-100, it becomes relevant to understand how this amine can cross the blood–brain barrier (BBB) and if this passage represents a limit for T1AM degradation (Figure 1).

The BBB consists of a specialized network of fenestrated capillaries in tight relation with astrocytes projecting to neurons, working as a physical barrier to the passage of humoral factors, including cholesterol and other lipids. Cholesterol flux is directed by lipoproteins but only the high-density lipoproteins can traverse the BBB. However, apolipoproteins, including Apo-E, are present in brain areas and in the cerebrospinal fluid (12). In addition, since endothelial cells express Apo-B100 (13), it can be argued apolipoproteins can be synthetized in brain areas. In this respect, astrocytes, which also express high levels of VLDL-R and LDL-R (14), have been indicated as the most likely candidate cell for apolipoprotein synthesis. Overall, the presence of apolipoproteins and of their receptors suggests that apolipoproteins may have functions in the brain. Consistently, evidence indicated lipoproteins can stimulate the cognitive function in the hippocampus and regulate energy balance in the hypothalamus (14).

3-Iodothyronamine is physiologically present in mouse thyroid, and it distributes in the gland following pharmacological administration. In the thyroid, the half-life of T1AM was the highest among the tissues analyzed (11).

Manni et al. (15) demonstrated that, following i.c.v. injection of T1AM, this amine was recovered in the systemic circulation, thus indicating it can pass but also exit the BBB. Interestingly, T1AM systemic bioavailability significantly increased when its oxidative metabolism was inhibited, i.e., mice pretreated with clorgyline, a mitochondrial MAO inhibitor. Consistently, Laurino et al. (16) demonstrated T1AM disappearance from the medium of cultured organotypic hippocampal slices associated with the contemporaneous accumulation of TA1 and that that, in mouse brain, T1AM pharmacologically administered is metabolized to TA1.

### T1AM Metabolism

For its chemical structure, T1AM has the requisites to be a substrate for amine oxidases including MAO (17), ubiquitous enzyme activities, and semicarbazide-sensitive amine oxidases (SSAOs), a heterogeneous class of enzymes localized on cell plasma membranes. Two isoforms of MAO have been cloned, i.e., MAO-A and MAO-B (18) with MAO-A mainly present in catecholaminergic and MAO-B in serotonergic and histaminergic neurons and astrocytes (19, 20).

Both MAO and SSAO are included within the pattern of non-microsomal phase-I metabolizing enzymes. Due to the absence of SSAOs in the brain, T1AM oxidative metabolism at this site can be carried on only by MAO. Lehmphul et al. (17) first demonstrated T1AM is a substrate for MAO-B opening to a new function for MAO-B (21). Furthermore, the involvement of MAO catalysis in T1AM degradation implies physiopathological and pharmacological consequences. In fact, the extent of T1AM degradation would depend on MAO-B tissue expression levels and on pathological conditions altering MAO-B promoter activity, including diabetes, inflammation, thyroid diseases, or aging. Secondarily, T1AM oxidation is expected to be modified by treatments including inhibitors of MAO-B as those used in the control of the early symptoms of Parkinson’s disease (18).

In addition, since MAO catalysis produces hydrogen peroxide, reactive aldehydes, and ammonia, the degradation of T1AM by MAO-B potentially alters the cell redox state (19). The impact of T1AM degradation on neuron redox state might represent a novel and interesting issue to explore.

Furthermore, the fact that in the thyroid a very high mitochondrial MAO-A, and low MAO-B, is present (22) might account for the long half-life of the amine in the gland.

### The Effects of T1AM Suggests the Amine Behaves as a Hormone or as a Neuromodulator

**Figure 2:**
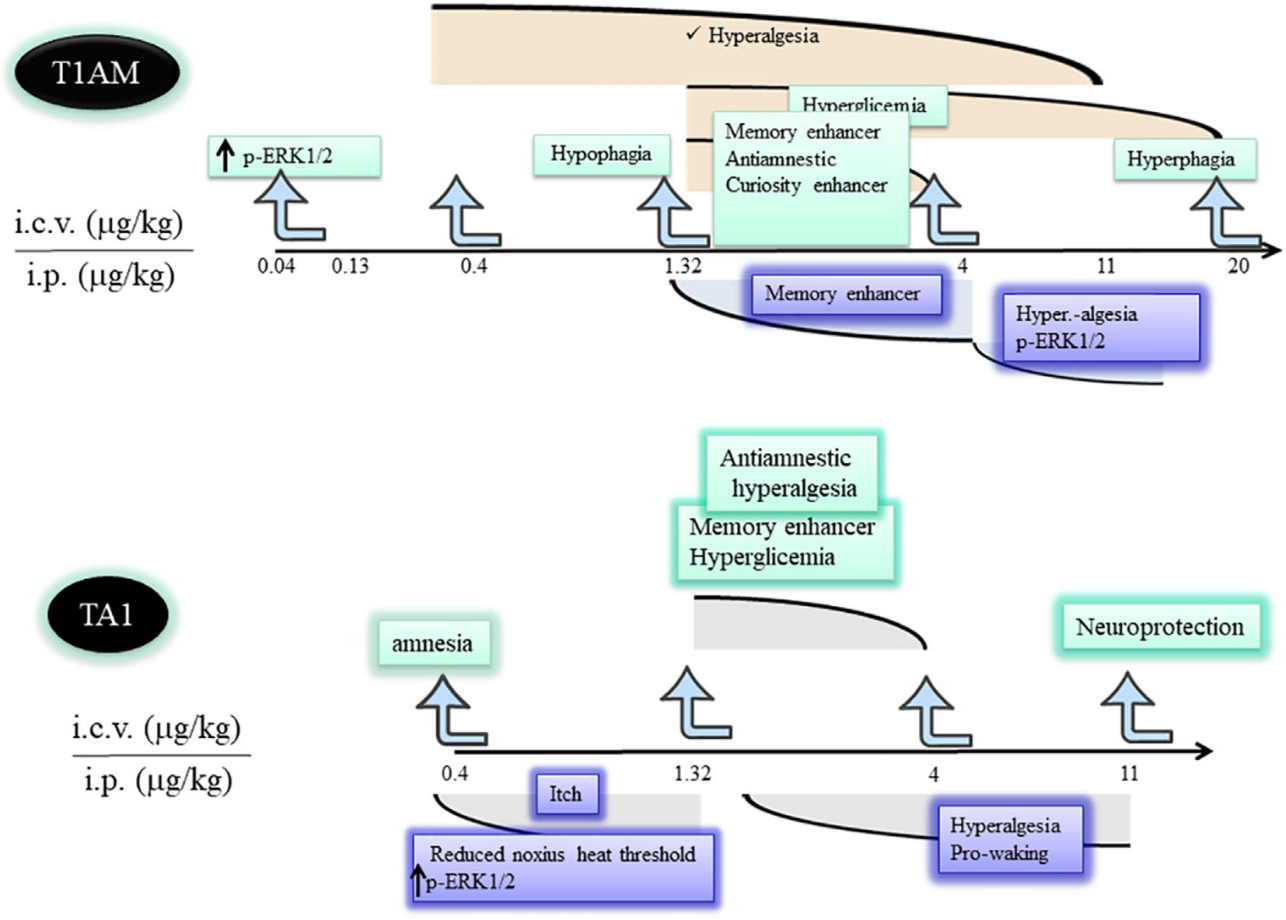
Differences and similarities between T1AM and TA1 pharmacological effects. T1AM and TA1 share pharmacological effects that are described by inverted dose–effect curves. The cartoon indicates similarities and differences among central effects of equimolar doses of T1AM and TA1. Abbreviations: T1AM, 3-iodothyronamine; TA1, 3-iodothyroacetic acid; T0AM, thyronamine; MAO, monoamine oxidases.

Experimental data indicate T1AM is a potent and effective modifier of the behavior and of the metabolism of rodents. Such effects onset within 15–30 min from amine administration and often show inverted U-shaped dose–effect curves. This characteristic suggests increasing T1AM doses (i) rapidly induce desensitization of a target(s) or (ii) recruit different targets controlling opposite effects. Whatever the mechanism, the inverted dose–effect curve indicates it is possible to predict the dose of T1AM that needs to be administered to observe a desired effect (Figure 2) and stresses the importance to study T1AM at its lowest effective dose.

Furthermore, the acute central effects of T1AM are followed by long-term effects based on the activation of gene transcription. Whether these effects are driven directly by T1AM, TA1, or indirectly by T1AM or TA1-induced release of neurotransmitters is unknown yet. Considering the current knowledge, histamine has been identified as one of the neurotransmitters involved in T1AM effects.

Histamine in the brain accumulates in mast cells, astrocytes (23), and in the few histaminergic neurons that are concentrated in the hypothalamus from which they project to most of the brain areas including the hippocampus and cortex (24). Neuronal histamine is implicated in memory, control of the sleep/wake cycle, feeding, thermoregulation, motility, and it is considered an endogenous neuroprotective mediator against excitotoxicity (25, 26). Histamine in the brain is mainly degraded by *N*-methyl transferase producing *N*-methylhistamine (27) which is, in turn, a good substrate for MAO-B. Consequently, histamine and T1AM may compete for MAO-B catalysis.

### Hypothalamic Effects of Pharmacologically Administered T1AM

Scanlan et al. (1) first described that high T1AM doses (50 mg/kg) induced a deep reduction of mice body temperature. Later, Doyle et al. (28) demonstrated T1AM rescued neurons from excitotoxicity by inducing hypothermia in an experimental model of cerebral ischemia. Some years later, James et al. (29) clarified that the hypothermia was secondary to increased heat dispersion caused by tail vasodilation (29) and indicated T1AM as a hypothalamic regulator of the temperature set point of warm–sensitive neurons. This mechanism might also explain the potent reduction of pain threshold to hot stimuli described after T1AM administration in mice (15).

Dhillo et al. (30) reported that T1AM reduced feeding of *ad libitum*-fed mice, and Ju et al. (31) described the amine shifted energy substrate utilization from glucose to lipids. Overall, this evidence contributed to delineate T1AM as a compound inducing a hypometabolic status. However, Manni et al. (15) unmasked the hyperphagic effect of the amine. In fact, the authors found that, at condition of fasting, i.c.v. injected low T1AM doses (1.3 µg/kg) still induced hypophagia and rapidly increased plasma glucose. Instead, injection of higher T1AM doses induced hyperphagia without raising plasma glycemia. Because of this, T1AM can be considered as a potent hypophagic compound able to counteract the fasting-induced tendency of mice to overfeeding. This effect is likely sustained by the increase of plasma glycemia. Consistently, at doses producing hyperphagia, plasma glycemia was not modified. In any case, the raise of plasma glycemia was a hypothalamic-mediated effect and it went in the opposite direction compared with the anti-hyperglycemic effect described for other TAs (32).

In addition, Manni et al. (15) demonstrated, for the first time, that T1AM oxidative metabolism was essential for T1AM hyperphagia. In fact, in mice pretreated with clorgyline, an inhibitor of MAO activity, hyperphagia disappeared while the hypophagic effect remained. Then, hypophagia could derived from the recognition of a high-affinity target (extracellular pathway), while hyperphagia depended on some products of T1AM oxidative deamination, including TA1 (intracellular pathway).

James et al. (29) reported that T1AM stimulated mice wakefulness when injected in the preoptic region, confirming the activity on hypothalamus. Furthermore, T1AM injected i.c.v. or systemically administered induced hyperalgesia to hot stimuli (33). The stimulation of peripheral sensory neurons translated in pERK1/2 of the interneurons of dorsal root ganglia (DRG), thus involving the spinothalamic pathway in the transmission of pain transmission (34).

### T1AM Activity at Hippocampus

Among the main interesting effects elicited by T1AM is its activity on memory circuits possibly indicating some effectiveness of the amine in diseases characterized by memory impairments (35).

3-Iodothyronamine given i.c.v. or systemically, increased pERK1/2 and pAKT levels in different brain areas, including the hippocampus and hypothalamus (33), it acutely produced an increase in mice exploratory activity of learning and reverted drug-induced amnesia (36). Furthermore, T1AM not only stimulated learning but also consolidated memory in the 24-h training session (33, 34) an effect consistent with pERK1/2, a master regulator of *de novo* synthesis of proteins necessary for memory consolidation. Interestingly, stimulation of learning was not observed in mice pretreated with clorgyline and with anti-histaminergic treatments. This evidence confirmed the role of MAO-B catalysis in the generation of a pro-learning compound and indicated the involvement of the histaminergic system (5, 34).

Interestingly, recent *in vitro* evidence obtained in isolated striatal slices gave indication on a receptor-mediated mechanism of T1AM. In particular, Zhang et al. (37) demonstrated T1AM, by activating TAAR1, promoted phosphorylation of thyroxine hydroxylase, thus prompting the synthesis of dopamine and its release. Interestingly enough, in the striatum, the main MAO isoform expressed is type B, which is the enzyme involved in T1AM (but also dopamine) degradation (20). Furthermore, other recent evidence also indicates that the release of dopamine in the striatum may be controlled by histamine released from the tuberomammillary nucleus, *via* H1 and H2 receptors (38). Overall, these results suggest T1AM might sustain dopamine synthesis and release acting at high-affinity targets (TAARs) and, indirectly, releasing histamine from the hypothalamic neurons. Both mechanisms might sustain an effectiveness of T1AM in basal ganglia diseases.

Furthermore, T1AM was also indicated as an activator of the serotoninergic type 1 subtype b receptor in virtue of heterodimerization of this receptor with TAAR1 (39), a finding that suggests possible antidepressant effectiveness of the amine.

However, the effects of T1AM on dopamine release and on the activation of the serotonin receptor need to be conclusively demonstrated in *in vivo* settings.

## Similarities and Differences in Behavioral Effects Induced by Pharmacological Administration of T1AM and TA1

If TA1 is T1AM active principle, then the pharmacological administration of TA1 should reproduce at least some of the effects described for T1AM. This assumption does not consider that TA1 pharmacologically administered might not have the same pharmacokinetic features of the acid produced *in situ* by T1AM deamination. Among these differences, TA1 pharmacokinetic does not include the activity of MAO.

Musilli et al. (40) studied the effect of TA1 i.c.v. injected on memory, pain, and plasma glycemia. This study indicated TA1 effect on memory was more composite than that of T1AM. In fact, at a low dose (0.4 µg/kg) the acid produced amnesia while at 1.32 and 4 µg/kg it stimulated learning without inducing memory consolidation. Interestingly, only the pro-learning effect was prevented by anti-histaminergic treatments. In particular, in the presence of zolantidine, TA1 turned out to be amnestic at all the doses tested. These differences in respect of T1AM effects on memory might relay on the pharmacokinetics of the acid but definitively prove that activation of the histaminergic system is triggered by TA1. However, TA1 present in the synaptic cleft may interact also with its own targets involved, for instance, in the amnestic effect. At the same pro-learning doses, TA1 induced hyperalgesia and hyperglycemia showing inverted U-shaped dose–effect curves (41) (Figure 2). Hyperalgesia and hyperglycemia were again modulated by anti-histaminergic drugs.

Hoefig et al. (42) reported that TA1 i.p. administered to mice at doses much higher (5 mg/kg) than those used by Manni et al. (15), did not induce hypophagia, hyperglycemia, and hypothermia thus confuting the hypothesis TA1 was the mediator of T1AM hypothalamic effects. Moreover, before concluding on this, it is noteworthy to recall that T1AM was not able to induce hyperglycemia when administered at high doses and the hypophagic effect of T1AM was found independent of MAO inhibition (15). Collectively, these data instead reinforced the hypothesis that T1AM and TA1, pharmacologically administered, have the possibility to activate their own target but that they share a common intracellular mechanism.

Laurino et al. (41) indicated that TA1, systemically administered, deeply affected mouse sensitivity to noxious and painful stimuli. As for T1AM, the reduction of pain threshold resulted to be dependent on histamine release and associated with ERK1/2 activation in DRG neurons.

3-Iodothryoacetic acid, as T1AM, systemically administered, increased mouse vigilance. In particular, TA1 stimulated the wakefulness of mice induced to sleep by a high ethanol dose (43), without interfering with the GABaergic transmission.

### TA1 Mechanism of Action: Possible Hypotheses

How TA1 activates the histaminergic system remains to be elucidated. In this respect, it is unlikely that TA1 works as a histamine type 3 receptor antagonist since it lacks the necessary chemical requisites to recognize the receptor. From our study, we can also exclude TA1 may interact at muscarinic and GABA-A receptors (43).

The study from Bellusci et al. (34) indicated the chemical requisite for activating the histaminergic system. In particular, synthetic analogs of T1AM, lacking the iodide atom and the diphenoxyl moiety, were reported to stimulate, as T1AM, memory acquisition and retention depending on MAO activity and on H1 receptor activation. Such results indicated that (i) the presence of the iodide and of the diphenoxyl moiety are not essential for activating the histaminergic system and (ii) the activation of the histaminergic system is secondary to intracellular accumulation of an acid compound that stimulates histamine release.

## Conclusion

Pharmacological evidence indicates T1AM activates signaling pathways in different brain areas including the hypothalamus and the hippocampus, reproducing in rodents’ behavioral effects typically mediated by these brain areas. Such behaviors are dependent on T1AM metabolism carried on by phase-I non-microsomal enzymes, producing TA1, and on the activation of the histaminergic system-mediated accumulation of TA1.

Furthermore, T1AM may also interact with high-affinity targets on plasma membrane, including TAAR1. Of these two mechanisms, one may prevail over the other depending on T1AM doses or on the effect studied.

The study of T1AM pharmacological profile allowed to reveal a novel and yet unexplored link between T1AM, MAO (MAO-B), and the histaminergic system.

## Author Contributions

EL and AL participated in performing experiments discussed in this review. In addition, they participated with LR in paper construction.

## Conflict of Interest Statement

The authors declare that the research was conducted in the absence of any commercial or financial relationships that could be construed as a potential conflict of interest. The handling Editor declared a past co-authorship with the authors AL and LR.
